# Sternocostoclavicular Hyperostosis: Positive Clinical and Radiological Response on Pamidronate

**DOI:** 10.3389/fendo.2021.621604

**Published:** 2021-02-18

**Authors:** Anne T. Leerling, Ana Navas Cañete, Ashna I. E. Ramautar, Natasha M. Appelman-Dijkstra, Elizabeth M. Winter

**Affiliations:** ^1^ Center for Bone Quality, Division of Endocrinology, Department of Internal Medicine, Leiden University Medical Center, Leiden, Netherlands; ^2^ Center for Bone Quality, Department of Radiology, Leiden University Medical Center, Leiden, Netherlands

**Keywords:** sternocostoclavicular hyperostosis, pamidronate, bisphosphonate, treatment, radiologic response, sclerosis, pain, SAPHO

## Abstract

**Background:**

Sternocostoclavicular hyperostosis (SCCH) is a rare disease, constituting a chronic sterile osteomyelitis with elevated bone turnover in the axial skeleton, causing pain and shoulder dysfunction. SCCH severely interferes with daily activities, work, and quality of life. SCCH has a relapse-remitting disease course, but inflammatory-induced sclerotic transformation in the affected area is slowly progressive. Here we present two patients with clinical and radiological diagnosis of SCCH treated with intravenous pamidronate, leading to clinical remission in both, but complete resolution of sclerosis in one of them, which is a novel finding in our experience.

**Case Presentation:**

Two adult female SCCH-patients presented with longstanding pain, swelling of the anterior chest wall, and compromised shoulder function. Subsequent single photon emission computed tomography-computed tomography (SPECT/CT) illustrated elevated bone activity and sclerosis in the SC region, with hyperostosis, confirming the diagnosis of SCCH. As symptoms in both patients were eventually refractory to standard painkillers such as non-steroidal anti-inflammatory drugs (NSAIDs), intravenous pamidronate treatment in 3-month cycles was started. Pamidronate was effective in reducing pain and improving shoulder function and also led to decreased bone turnover on skeletal scintigraphy. Sclerosis in the first patient persisted. In the second patient, however, a complete resolution of sclerosis was observed.

**Conclusions:**

SCCH remains a rare bone disorder for which no evidence-based therapies are yet available. While disease burden is high, SCCH lacks recognition and is often diagnosed long after symptomatic presentation. As for the cases in this report, pamidronate was successful in reducing symptoms, and in the second case even led to regression of sclerotic changes on CT-imaging.

## Background

Sternocostoclavicular hyperostosis (SCCH) is a rare chronic inflammatory disease, comprising a sterile osteomyelitis of the axial skeleton mainly affecting the sternocostoclavicular region. Here, an inflammatory cascade leads to increased local bone turnover, favoring bone formation (1–3). SCCH can be a part of SAPHO syndrome encompassing synovitis, acne, pustolosis, hyperostosis, and osteitis, but is also referred to as a separate clinical entity (4, 5). Together with chronic recurrent osteomyelitis (CRMO) and diffuse sclerosing osteomyelitis (DSO), SCCH belongs to the spectrum of chronic non-bacterial osteomyelitis (CNO).

SCCH usually affects patients in midlife, and a clear predisposition for the female gender has been described in recent case series (5). Clinical manifestations include a painful swelling of the sternum, ribs and clavicles and impaired mobility of the shoulder girdle (1). As a consequence, patients experience serious interference with their quality of life (5). SCCH typically has a chronic nature, with a relapse-remitting course and varying rates of bone turnover on scintigraphy, though spontaneous remission can be seen (6). Inflammatory-induced changes such as sclerosis and hyperostosis are usually slowly progressive (1, 7). Left untreated, SCCH may lead to permanent degenerative changes of the adjacent joints and secondary ossification of soft tissue (1, 8), which may further compromise shoulder girdle function. Although there is no established treatment for SCCH, pain control can sometimes be attained with NSAIDs (3). Other medications used are biologicals (9–11) or intravenous bisphosphonates, mostly pamidronate (12–16), which decrease inflammation or both inflammation and bone turnover, respectively.

Here we present the disease course in two SCCH-patients treated with intravenous pamidronate.

## Case Presentation (1)

A 58-year-old female patient was referred to our center with longstanding shoulder pain. Medical history was positive for pustulosis palmoplantaris, which was adequately controlled with topical steroids. Family medical history was positive for autoimmune disease, with a sister suffering from ulcerative colitis and a brother with Sjogren’s Syndrome. The patient quit smoking 7 cigarettes per day (approximately 14 packyears in total) 2 months before consultation.

**Figure 1:**
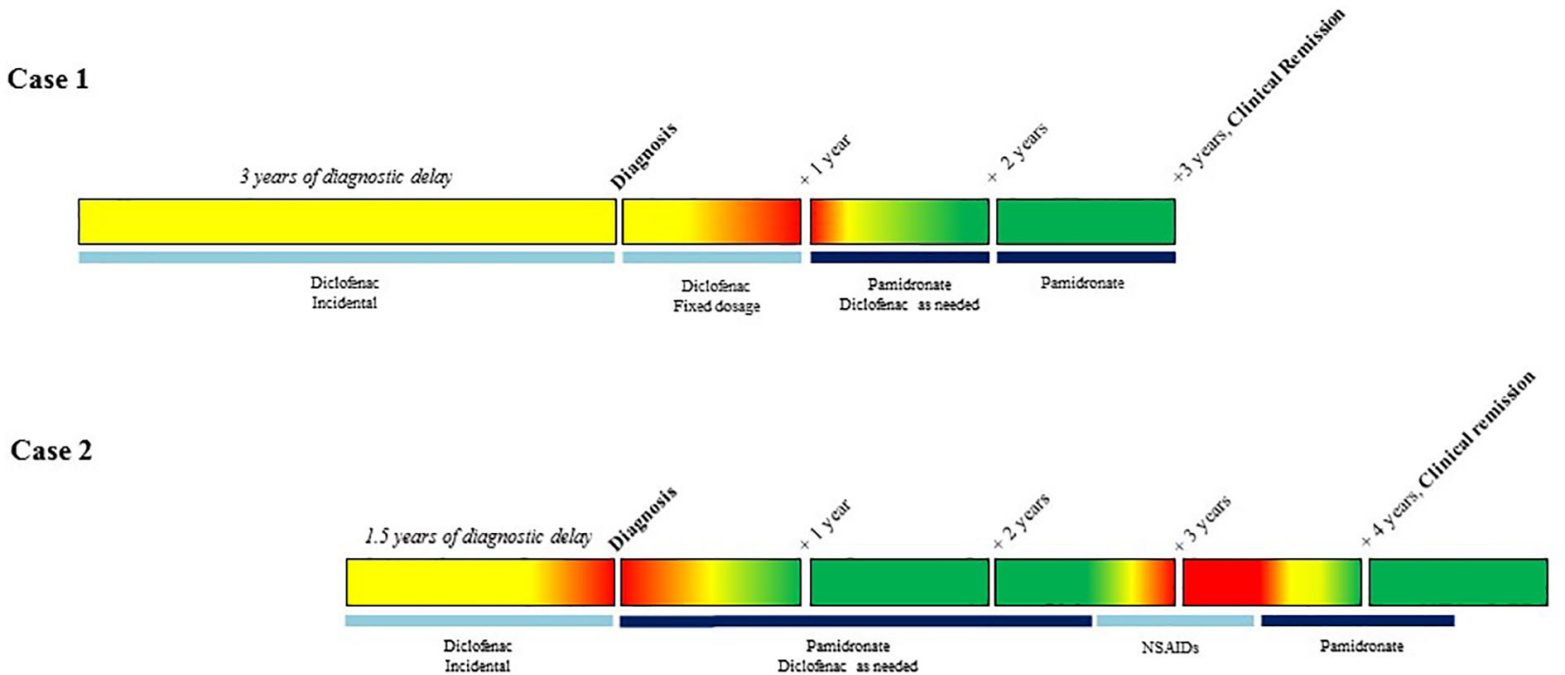
Overview of treatment status and clinical reports of pain. Legend: 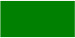 No pain, 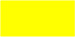 Medium pain, 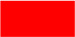 Severe pain.

**Figure 2:**
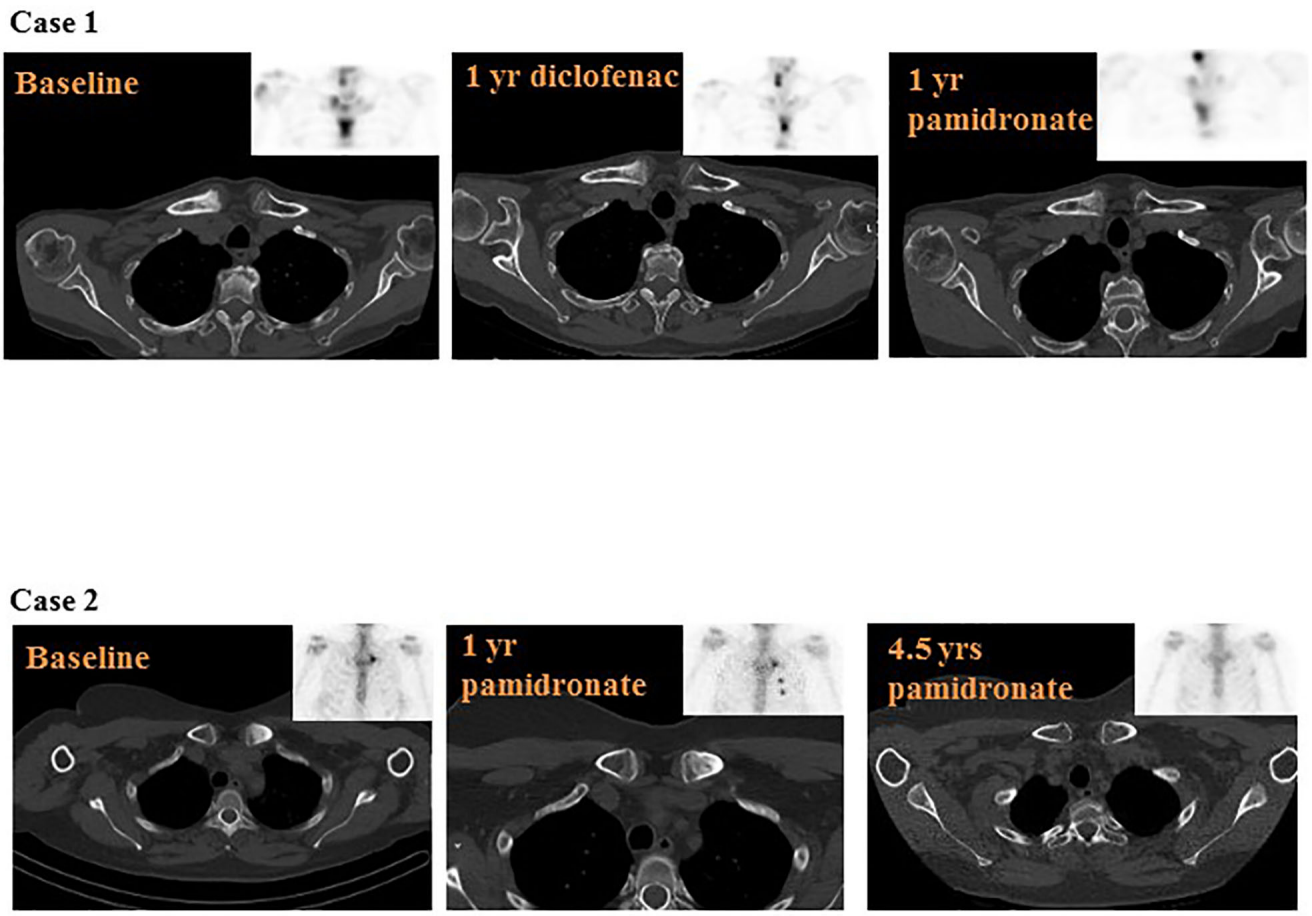
Overview of disease course on computed tomography (CT)-scan and skeletal scintigraphy. Legend: Case 1: Baseline: diffuse sclerosis of the right clavicular end with increased bone turnover, and increased bone turnover of the manubrium and proximal corpus sterni. 1 year diclofenac: persistent sclerosis and increased bone turnover of the right clavicular end and persistent increase of bone turnover of the manubrium and proximal corpus sterni, slightly reduced compared to baseline. 1 year pamidronate: unchanged sclerosis and reduction of bone turnover of the right clavicular end, the manubrium and proximal corpus sterni, compared to baseline. Case 2: Baseline: sclerotic changes of the left clavicular end with elevated bone turnover. 1 year pamidronate: slightly reduced sclerosis of the left clavicular end and slightly reduced bone turnover. The increased uptake of the 3^rd^ and 4^th^ rib was caused by two costal fractures. 4.5 years pamidronate: almost complete resolution of sclerosis, leaving a minimal rest, and complete normalization of bone turnover.

Shoulder pain in rest, numerical score (NRS) 4, and during movement (NRS 8), was present for 3 years (**Figure 1**), and was previously diagnosed as tendinopathy with impingement. Despite conservative therapy, pain and impaired mobility persisted, and were later accompanied by redness and swelling of the sternum. Physical examination showed a painful sternal swelling with visible erythema. Laboratory results were normal including inflammatory markers, except for a mild vitamin D deficiency of 44 nmol/L, ref: >50 nmol/L, for which supplements were prescribed. Creatinine clearance was adequate before the start of treatment and remained so during further treatment course, ranging 67–70 µmol/L, ref: 49–90 µmol/L. Subsequent skeletal scintigraphy with single position emission computed tomography-computed tomography (SPECT/CT) demonstrated increased bone turnover of the right clavicular end, the manubrium and proximal corpus sterni, with sclerosis, and hyperostosis (**Figure 2**). Integrating clinical symptoms with radiological findings, the differential diagnosis of SCCH in isolated form was considered. And, due to the presence of pustulosis palmoplantaris this SCCH was considered as part of SAPHO syndrome.

NSAIDs were started, with diclofenac in a fixed dose of 75 mg a day. After 6 months, pain was scored as 3 and the shoulder still retained full range of motion (ROM). However, after 1 year of diclofenac treatment, pain increased to 6 on NRS and shoulder ROM was limited to 100 (left) and 110 (right) degrees in active abduction. Repeated radiological and nuclear imaging showed persistent sclerosis and increase of bone turnover of the right clavicular end, though slightly reduced compared to baseline (**Figure 2**). Given the worsening of the clinical course and conform treatment protocol at our center, therapy with intravenous pamidronate in a regimen of 3 consecutive infusion days dosed 15-30-30 mg in 3 monthly intervals was started (**Figure 1**). Additional diclofenac was recommended. Shortly after the first cycle of pamidronate, the patient reported significant improvement of symptoms with a quickly reclaimed free range of motion (FROM). After 1 year of pamidronate (four cycles), the patient reported further reduction of pain (NRS 4) with no need for additional diclofenac, and persistent FROM. As for radiological disease course, scintigraphy showed reduction of bone turnover of the right clavicular end compared to baseline with unchanged sclerotic changes on CT (**Figure 2**). Due to the good clinical response and the absence of adverse events, pamidronate was continued in a 3-day regimen of 30 mg per day in 3 monthly intervals. After 2 years of pamidronate-treatment, clinical remission was reached on grounds of the patient reporting durable pain control and full shoulder mobility, and pamidronate was stopped.

## Case Presentation (2)

A 49-year old female patient, with a medical history of asthma, M. Dupuytren, and migraine presented at our center with swelling and pain in the left shoulder since 1.5 years. Medication included vitamin D supplementation, omeprazole, desloratadine and sporadic Ventolin. She smoked incidentally (one cigarette per 3 months, which she quitted at year 3 of therapy). Family medical history was positive for diabetes mellitus type 1 in first- and second-degree relatives. The symptoms were previously ascribed to a bursitis and treated with two corticosteroid injections. Pain, however, persisted with a numerical score of 8 and significantly interfered with daily functioning and sleeping despite diclofenac. Physical examination revealed a painful swelling of the left sterno-clavicular region, with visible erythema and warmth. Laboratory results only showed a minimally elevated C-reactive protein of 5.4 mg/L, ref < 5 mg/L and an alkaline phosphatase of 114 U/L, ref 20–140 U/L, later accompanied by an elevated gamma-glutamyl transferase (ranging 49–63 U/L, ref < 25 U/L), altogether suspicious for hepatic origin. Creatinine clearance was and remained within reference range treatment course, ranging 65–74 µmol/L, ref 49–90 µmol/L. A CT scan of the chest-wall displayed a non-specific bilateral linear calcification of costa 1 and unilateral sclerosis of the left proximal clavicula. This area of clavicular sclerosis did not involve the complete subchondral articular surface of the clavicula and the joint space (sternoclavicular joint) was preserved, hence the diagnosis of reactive sclerosis due to degenerative joint disease was unlikely. Preliminary calcification of the left costoclavicular ligament was also detected. Skeletal scintigraphy showed increased uptake of left proximal clavicula at the level of the sclerotic area, but not at the sternal side in sternoclavicular transition (**Figure 2**). Together, the clinical picture with this radiological pattern confirmed the diagnosis of SCCH.

As pain was inadequately controlled with daily NSAIDs, the patient was started on intravenous pamidronate in a 5-day regimen of 15 mg per day, in 3-month intervals (**Figure 1**). Diclofenac was recommended when needed and the patient was advised to quit smoking. Therapy was effective in reducing pain, erythema and warmth of the sternoclavicular region. After 1 year of treatment, VAS score decreased to 6, range of motion was fully restored and CT imaging as well as skeletal scintigraphy showcased a slight reduction in sclerosis as well as bone activity compared to baseline imaging (**Figure 2**). Pamidronate was temporarily stopped after 2.5 years of treatment but restarted in a modified 4-day regimen 15 mg per day in 4 monthly intervals, due to recurrence of severe pain. After 4.5 years since the start of pamidronate treatment, remission was established on the grounds of absence of pain (NRS 0), restored shoulder function (FROM), no need for complementary diclofenac, and complete normalization of bone activity on skeleton scintigraphy (**Figure 2**). Most remarkably, CT-imaging showcased an almost complete resolution of former sclerosis, leaving only a minimal rest.

## Discussion and Conclusions

SCCH is a rare inflammatory bone disorder, of which the precise underlying mechanisms remain yet to be determined. Low awareness among physicians, consequent diagnostic and therapeutic delay, and associated decreased quality of life and psychological wellbeing of patients, all contribute to a challenging care process (5, 17).

The nomenclature surrounding SCCH is complex and potentially confusing. Chronic non-bacterial osteomyelitis (CNO) functions as the umbrella term covering SCCH and its related clinical entities. Among these are chronic recurrent multifocal osteomyelitis (CRMO), a predominately pediatric condition typically localized in the metaphyses of long bones of the extremities and the medial clavicles, in addition to other less frequent locations such as vertebral bodies, pelvis, ribs, and mandible (18), and diffuse sclerotic osteomyelitis (DSO), a disease of the mandible in specific (19). Also belonging to the CNO spectrum is the earlier mentioned SAPHO syndrome, of which there is ongoing discussion whether it is the adult version of CRMO (18). Coming to, SCCH, the semantic complexity increases with literature presenting SCCH as being a part of SAPHO syndrome as well as SCCH in isolated form (4, 5). In this paper, we deliberately use both definitions of SCCH, as we perceive that many patients (such as the patient in case 2) with characterized SCCH do not match the rest of the SAPHO acronym. In addition, signs of systemic inflammation such as elevated erythrocyte sedimentation rate are frequently seen in SAPHO syndrome (18), but are usually absent in isolated SCCH (6). In the two cases presented, though, this distinction was not present. The use of SCCH as a separate term, with or without other SAPHO manifestations present, can also be useful when deciding on therapeutic strategy. Bisphosphonates such as pamidronate have shown to improve bone, but not cutaneous manifestations of SAPHO syndrome (20), qualifying them for the treatment of isolated SCCH, illustrated in case 2, or for the SCCH component of SAPHO causing the highest disease burden, such as in case 1. For patients suffering from the wider symptomatology of SAPHO syndrome, anti-inflammatory drugs such as methotrexate and/or biologicals may be an alternative treatment choice (18, 20).

The diagnosis of SCCH can thus be made by the combination of clinical and radiological characteristics, and after the evaluation of manifestations characteristic for an overarching SAPHO syndrome. As briefly mentioned before, serological markers, inflammatory parameters and bone turnover markers are usually normal in SCCH, and used to exclude further differential diagnoses, such as infection, neoplasm or spondyloarthropathies (1, 6). In the two presented cases, diagnosis of SCCH was, in line with the trend in literature, only made after years of pain and shoulder dysfunction, constructing significant burden and decreased quality of life. Radiographically, both cases presented with features consistent with SCCH. SCCH is characterized by sclerosis and hyperostosis typically in the proximal clavicles, sternum and upper ribs, perchance accompanied by secondary degeneration and bony erosions of the sternoclavicular joint (1, 21). These findings are better and earlier seen on computed tomography (CT) than on conventional radiography (21), however clear radiographical criteria are lacking. On skeletal scintigraphy, increased uptake in the sternoclavicular joint area, the costal cartilages of the first and second ribs, and the manubrium sterni is highly suggestive of SCCH and considered a hallmark of disease, and in its symmetrical form referred to as the “bull head sign” (18, 22). As for (full body) MRI, this imaging modality is preferred in (pediatric) CRMO patients due to its sensitivity to detect bone marrow edema, arthritis/synovitis (a common manifestation of this disease) and so as to evade radiation exposure (23). In SCCH, however, bone marrow edema is not commonly present, especially not in later stages of the disease. Since the majority of patients present with significant diagnostic delay (24), at which point the sclerosis and hyperostosis are the more typical characteristics, CT proves an adequate imaging modality, and combining the scan with full body scintigraphy enables the detection of subclinical lesions as well (18, 21, 25).

As there are no disease tracking biomarkers nor established therapy for SCCH, treatment decisions are primarily made on empirical data and expert opinion. Since over two decades patients with active isolated SCCH (and thus suffering mostly from the bone manifestations) and refractory to NSAIDs are treated with intravenous pamidronate with satisfactory effect on pain and shoulder mobility. The rationale for treatment with bisphosphonates evolves from their inhibitory effect on bone turnover, especially at sites where this turnover is elevated (26). For SCCH patients, this increased turnover presumably lies at the root of pain and herewith suggests the potential effect of bisphosphonates. In other metabolic bone diseases characterized by local increase in bone turnover such as Paget’s disease of the bone, this effect has been observed as well (27, 28). A second mechanism of action might derive from the anti-inflammatory properties of bisphosphonates due to their inhibition of Farsenyl Pyrophosphate dependent macrophages *via* the melavonate pathway (29), and them decreasing the level of circulating gamma/delta T cells, a subset of CD3+ T cells (30). Additionally, pamidronate in specific is known to cut down on lymphocyte proliferation and lymphocyte/monocyte interaction (31, 32).

Should patients be refractory to bisphosphonate therapy, alternative treatment options are (limitedly) at hand including the afore mentioned biologicals, which have shown their effect on both skin and bone lesions in SAPHO syndrome (18). There is some data on the positive effect of denosumab in DSO patients refractory to bisphosphonates (33). However, experience is limited to single cases, denosumab lacks the specific distribution to areas of increased bone turnover, and is associated with risk of rebound in osteoporosis (34).

Pamidronate confirmed its favorable clinical effect in the two cases presented; pain and compromised shoulder function significantly decreased, and in both patients we were able to stop treatment after several years. As for radiological features, the rate of local bone turnover typically fluctuates, following the relapse remitting character of disease course. Structural change, however, is commonly progressive, developing from enthesopathy of the costoclavicular ligament with erosion and increased bone turnover, into increased local sclerosis, followed by further hyperostosis and involvement of soft tissue. The latter often results in continuous pain due to secondary degenerative changes, altogether leading to further disease burden and impaired quality of life (5, 17, 21). In our two cases, we observed a local reduction in bone turnover on scintigraphy in both, with persistent sclerosis in the first, and, remarkably, resolution of sclerosis in the second. For CRMO, full resolution of lesions on MRI in children after both NSAID and pamidronate therapy has been described earlier (35–37), just like for DSO an improvement of structural bone changes after pamidronate therapy has been reported (38, 39). A recent randomized study on the effect of pamidronate in CNO patients in general did report significant improvement on radiological disease activity, but not on chronic inflammatory changes (40). Thus, for SCCH the potential of full resolution of sclerosis has, to our knowledge, not been firmly established yet. The observation of sclerosis resolving in our patient is therefore relevant, as the regression plausibly lowers the chance of secondary degenerative transformation, and therefore may prevent permanent disability. Hence, considering this a potential treatment outcome, the grounds on which pamidronate treatment is given for SCCH are slightly strengthened.

However, the implications of our findings need to be approached with caution. Firstly, it cannot be undoubtedly stated that the resolution of sclerosis in patient 2 is a direct result of pamidronate-treatment. The possibility of spontaneous improvement remains, and can only sufficiently be rejected when the treatment modality of pamidronate is systematically researched. Nonetheless, the quick and major clinical response of longstanding complaints in the presented cases, topped by the radiological regression of sclerosis in the second, does suggest a relation. Besides, SCCH being a rare and poorly recognized disease, only further emphasizes the need for more standardized studies.

In conclusion, this report contained two cases with typical presentation of SCCH: significant diagnostic delay with a complex diagnostic process, fluctuating disease course with positive effect of pamidronate-treatment. In case 1, we observed a decrease in pain and shoulder complaints, and reduced uptake on bone scintigraphy, whereas structural radiological changes including sclerosis persevered, in line with typical disease course. The resolution of sclerosis on top of the similar clinical effect in case 2 was, on the contrary, novel. This finding suggests that pamidronate-treatment might not only be effective in reducing pain and shoulder dysfunction, but may even reverse structural tissue transformation and therefore possibly restrain degenerative changes in the SCCH-affected area.

## Data Availability Statement

The raw data supporting the conclusions of this article will be made available by the authors, without undue reservation.

## Ethics Statement

Ethical review and approval was not required for the study on human participants in accordance with the local legislation and institutional requirements. The patients/participants provided their written informed consent to participate in this study. Written informed consent was obtained from the individual(s) for the publication of any potentially identifiable images or data included in this article. Written consent was acquired from both patients to establish and publish this report.

## Author Contributions

AL and EW collected and interpreted the data concerning the two cases, and drafted the report. AC assessed, described, and interpreted the radiological and nuclear imaging content of the two cases. AR and NA-D revised the report critically for intellectual content. EW supervised AL and also revised the report critically. All authors contributed to the article and approved the submitted version.

## Conflict of Interest

The authors declare that the research was conducted in the absence of any commercial or financial relationships that could be construed as a potential conflict of interest.

## Abbreviations

CNO, chronic non-bacterial osteomyelitis; CRMO, chronic recurrent multifocal osteomyelitis; DSO, diffuse sclerosing osteomyelitis; FROM, free range of motion; NRS, numerical rating scale; NSAIDs, non-steroidal anti-inflammatory drugs; PPP, pustolosis palmoplantaris; ROM, range of motion; SAPHO, synovitis, acne, pustolosis, hyperostosis, and osteitis; SC, sternoclavicular; SCCH, sternocostoclavicular hyperostosis; SPECT/CT, Single proton emission computed tomography.

## References

[1] Carroll MB. Sternocostoclavicular hyperostosis: a review. Ther Adv Musculoskelet Dis (2011) 3(2):101–10. 10.1177/1759720X11398333 PMC3382681 22870470

[2] Saghafi M Henderson MJ Buchanan WW. Sternocostoclavicular hyperostosis. Semin Arthritis Rheumatol (1993) 22(4):215–23. 10.1016/0049-0172(93)80070-V 8484129

[3] Kalke S Perera SD Patel ND Gordon TE Dasgupta B. The sternoclavicular syndrome: experience from a district general hospital and results of a national postal survey. Rheumatology (Oxford) (2001) 40(2):170–7. 10.1093/rheumatology/40.2.170 11257153

[4] Chamot AM Benhamou CL Kahn MF Beraneck L Kaplan G Prost A. Acne-pustulosis-hyperostosis-osteitis syndrome. Results of a national survey. 85 cases. Rev Rhum Mal Osteoartic (1987) 54(3):187–96. 2954204

[5] van der Kloot WA Chotkan SA Kaptein AA Hamdy NA. Diagnostic delay in sternocostoclavicular hyperostosis: impact on various aspects of quality of life. Arthritis Care Res (Hoboken) (2010) 62(2):251–7. 10.1002/acr.20075 20191525

[6] Nungu S Olerud C Rehnberg L. Sternocostoclavicular hyperostosis. Presentation and long-term follow-up of three cases. Ups J Med Sci (1992) 97(2):177–82. 10.3109/03009739209179294 1471317

[7] Dihlmann W Dihlmann SW. Acquired hyperostosis syndrome: spectrum of manifestations at the sternocostoclavicular region. Radiologic evaluation of 34 cases. Clin Rheumatol (1991) 10(3):250–63. 10.1007/BF02208686 1790633

[8] Fritz P Baldauf G Wilke HJ Reitter I. Sternocostoclavicular hyperostosis: its progression and radiological features. A study of 12 cases. Ann Rheum Dis (1992) 51(5):658–64. 10.1136/ard.51.5.658 PMC1005702 1616334

[9] Ben Abdelghani K Dran DG Gottenberg JE Morel J Sibilia J Combe B. Tumor necrosis factor-alpha blockers in SAPHO syndrome. J Rheumatol (2010) 37(8):1699–704. 10.3899/jrheum.091086 20472920

[10] Garcovich S Amelia R Magarelli N Valenza V Amerio P. Long-term treatment of severe SAPHO syndrome with adalimumab: case report and a review of the literature. Am J Clin Dermatol (2012) 13(1):55–9. 10.2165/11593250-000000000-00000 22007948

[11] Hampton SL Youssef H. Successful treatment of resistant SAPHO syndrome with anti-TNF therapy. BMJ Case Rep (2013) 2013. 10.1136/bcr-2012-007161 PMC3603525 23355559

[12] Marshall H Bromilow J Thomas AL Arden NK. Pamidronate: a novel treatment for the SAPHO syndrome? Rheumatology (Oxford) (2002) 41(2):231–3. 10.1093/rheumatology/41.2.231-a 11886977

[13] Colina M La Corte R Trotta F. Sustained remission of SAPHO syndrome with pamidronate: a follow-up of fourteen cases and a review of the literature. Clin Exp Rheumatol (2009) 27(1):112–5. 19327238

[14] Delattre E Guillot X Godfrin-Valnet M Prati C Wendling D. SAPHO syndrome treatment with intravenous pamidronate. Retrospective study of 22 patients. Joint Bone Spine (2014) 81(5):456–8. 10.1016/j.jbspin.2014.01.017 24561020

[15] Yachoui R Kreidy M Parker BJ. Treatment-Refractory Sternocostoclavicular Hyperostosis. Clin Med Res (2017) 15(1–2):37–40. 10.3121/cmr.2017.1352 28751466 PMC5573521

[16] Ringe JD Faber H Farahmand P. Rapid pain relief and remission of sternocostoclavicular hyperostosis after intravenous ibandronate therapy. J Bone Miner Metab (2006) 24(1):87–93. 10.1007/s00774-005-0651-2 16369904

[17] van der Kloot WA Hamdy NA Hafkemeijer LC den Dulk FM Chotkan SA van Emmerik AA. The psychological burden of an initially unexplained illness: patients with sternocostoclavicular hyperostosis before and after delayed diagnosis. Health Qual Life Outcomes (2010) 8:97. 10.1186/1477-7525-8-97 20828391 PMC2954978

[18] Rukavina I. SAPHO syndrome: a review. J Child Orthop (2015) 9(1):19–27. 10.1007/s11832-014-0627-7 25585872 PMC4340847

[19] Mari A Morla A Melero M Schiavone R Rodriguez J. Diffuse sclerosing osteomyelitis (DSO) of the mandible in SAPHO syndrome: a novel approach with anti-TNF therapy. Systematic review. J Craniomaxillofac Surg (2014) 42(8):1990–6. 10.1016/j.jcms.2014.09.004 25441866

[20] Aljuhani F Tournadre A Tatar Z Couderc M Mathieu S Malochet-Guinamand S. The SAPHO syndrome: a single-center study of 41 adult patients. J Rheumatol (2015) 42(2):329–34. 10.3899/jrheum.140342 25512472

[21] Depasquale R Kumar N Lalam RK Tins BJ Tyrrell PN Singh J. SAPHO: What radiologists should know. Clin Radiol (2012) 67(3):195–206. 10.1016/j.crad.2011.08.014 21939963

[22] Freyschmidt J Sternberg A. The bullhead sign: scintigraphic pattern of sternocostoclavicular hyperostosis and pustulotic arthroosteitis. Eur Radiol (1998) 8(5):807–12. 10.1007/s003300050476 9601969

[23] Morbach H Schneider P Schwarz T Hofmann C Raab P Neubauer H. Comparison of magnetic resonance imaging and 99mTechnetium-labelled methylene diphosphonate bone scintigraphy in the initial assessment of chronic non-bacterial osteomyelitis of childhood and adolescents. Clin Exp Rheumatol (2012) 30(4):578–82. 22765947

[24] Ramautar A Appelman-Dijkstra N Lakerveld S Valkema P Snel M Schroijen M. Clinical features of Sternocostoclavicular Hyperostosis: a large Single Center Dutch Cohort. J Bone Miner Res (2018) 32 (suppl 1).

[25] Buch K Thuesen ACB Brons C Schwarz P. Chronic Non-bacterial Osteomyelitis: A Review. Calcif Tissue Int (2019) 104(5):544–53. 10.1007/s00223-018-0495-0 30456556

[26] Rogers MJ Crockett JC Coxon FP Monkkonen J. Biochemical and molecular mechanisms of action of bisphosphonates. Bone (2011) 49(1):34–41. 10.1016/j.bone.2010.11.008 21111853

[27] Vasireddy S Talwalkar A Miller H Mehan R Swinson DR. Patterns of pain in Paget’s disease of bone and their outcomes on treatment with pamidronate. Clin Rheumatol (2003) 22(6):376–80. 10.1007/s10067-003-0762-x 14677009

[28] Corral-Gudino L Tan AJ Del Pino-Montes J Ralston SH. Bisphosphonates for Paget’s disease of bone in adults. Cochrane Database Syst Rev (2017) 12:CD004956. 10.1002/14651858.CD004956.pub3 29192423 PMC6486234

[29] Rogers MJ. From molds and macrophages to mevalonate: a decade of progress in understanding the molecular mode of action of bisphosphonates. Calcif Tissue Int (2004) 75(6):451–61. 10.1007/s00223-004-0024-1 15332174

[30] Rossini M Adami S Viapiana O Fracassi E Ortolani R Vella A. Long-term effects of amino-bisphosphonates on circulating gammadelta T cells. Calcif Tissue Int (2012) 91(6):395–9. 10.1007/s00223-012-9647-9 23052225

[31] de Vries E van der Weij JP van der Veen CJ van Paassen HC Jager MJ Sleeboom HP. In vitro effect of (3-amino-1-hydroxypropylidene)-1,1-bisphosphonic acid (APD) on the function of mononuclear phagocytes in lymphocyte proliferation. Immunology (1982) 47(1):157–63. PMC1555519 6214497

[32] Bijvoet OL Frijlink WB Jie K van der Linden H Meijer CJ Mulder H. APD in Paget’s disease of bone. Role of the mononuclear phagocyte system? Arthritis Rheum (1980) 23(10):1193–204. 10.1002/art.1780231018 6448605

[33] Hallmer F Korduner M Moystad A Bjornland T. Treatment of diffuse sclerosing osteomyelitis of the jaw with denosumab shows remarkable results-A report of two cases. Clin Case Rep (2018) 6(12):2434–7. 10.1002/ccr3.1894 PMC6293128 30564344

[34] Lamy O Stoll D Aubry-Rozier B Rodriguez EG. Stopping Denosumab. Curr Osteoporos Rep (2019) 17(1):8–15. 10.1007/s11914-019-00502-4 30659428

[35] Miettunen PM Wei X Kaura D Reslan WA Aguirre AN Kellner JD. Dramatic pain relief and resolution of bone inflammation following pamidronate in 9 pediatric patients with persistent chronic recurrent multifocal osteomyelitis (CRMO). Pediatr Rheumatol Online J (2009) 7:2. 10.1186/1546-0096-7-2 19138427 PMC2631594

[36] Roderick M Shah R Finn A Ramanan AV. Efficacy of pamidronate therapy in children with chronic non-bacterial osteitis: disease activity assessment by whole body magnetic resonance imaging. Rheumatology (Oxford) (2014) 53(11):1973–6. 10.1093/rheumatology/keu226 24899664

[37] Berkowitz YJ Greenwood SJ Cribb G Davies K Cassar-Pullicino VN. Complete resolution and remodeling of chronic recurrent multifocal osteomyelitis on MRI and radiographs. Skeletal Radiol (2018) 47(4):563–8. 10.1007/s00256-017-2812-5 29124297

[38] Li X Jia K An J Zhang Y. Application of pamidronate disodium for the treatment of diffuse sclerosing osteomyelitis of the mandible: a clinical study. Oral Surg Oral Med Oral Pathol Oral Radiol (2020) 130(6):616–24. 10.1016/j.oooo.2020.06.023 32771415

[39] Urade M Noguchi K Takaoka K Moridera K Kishimoto H. Diffuse sclerosing osteomyelitis of the mandible successfully treated with pamidronate: a long-term follow-up report. Oral Surg Oral Med Oral Pathol Oral Radiol (2012) 114(4):e9–12. 10.1016/j.oooo.2012.02.017 22771405

[40] Andreasen CM Jurik AG Deleuran BW Horn HC Folkmar TB Herlin T. Pamidronate in chronic non-bacterial osteomyelitis: a randomized, double-blinded, placebo-controlled pilot trial. Scand J Rheumatol (2020) 49(4):312–22. 10.1080/03009742.2020.1724324 32484386

